# Supplementary material for: “Addressing the bigger picture”: A qualitative study of internal medicine patients’ perspectives on social needs data collection and use
Source: PLoS One. 2023 Jun 7;18(6):e0285795. doi: 10.1371/journal.pone.0285795 (PMC10246844; doi:10.1371/journal.pone.0285795)
Supplement: S1 File — (DOCX) [file pone.0285795.s002.docx]

**S2 File. Sociodemographic and Social Needs Questionnaire**

**Sociodemographics**

1. **Were you born in Canada?** (Yes/No)

If no, what year did you arrive in Canada? ___________

1. **Do you identify as First Nations, Métis and/or Inuk/Inuit**?
   - Yes, First Nations
   - Yes, Métis
   - Yes, Inuk/Inuit
   - No
2. **We now know that people of different races do not have significantly different genetics. But our race still has important consequences, including how we are treated by different individuals and institutions. Which race category best describes you? Check all that apply:**

- Black (African, Afro-Caribbean, African Canadian descent)
- East/Southeast Asian (Chinese, Korean, Japanese, Taiwanese descent or Filipino, Vietnamese, Cambodian, Thai, Indonesian, other Southeast Asian descent)
- Indigenous (First Nations, Metis, Inuk/Inuit descent)
- Latino (Latin American, Hispanic descent)
- Middle Eastern- Arab, Persian, West Asian descent (e.g., Afghan, Egyptian, Iranian, Lebanese, Turkish, Kurdish)
- South Asian- South Asian descent (e.g., East Indian, Pakistani, Bangladeshi, Sri Lankan, Indo-Caribbean)
- White- European descent
- Another race category: ________________

1. **In general, do you experience any of the following due to a physical, mental, or emotional condition? (*Select all that apply)***
   - Difficulty seeing
   - Difficulty hearing
   - Difficulty walking or climbing
   - Difficulty remembering or with concentration
   - Difficulty with self-care
   - Difficulty with communicating
   - None of the above
2. **What was your sex assigned at birth?**
   - Male
   - Female
   - Intersex
3. **What is your current gender identity?**
   - Female
   - Male
   - Transgender Male/Trans Man/Female-to-Male (FTM)
   - Transgender Female/Trans Woman/Male-to-Female (MTF)
   - Gender fluid or Gender nonbinary
   - Two-Spirit
   - Another (Specify)__________
4. **Which best describes your sexual orientation?**
   - Heterosexual (“straight”, male/female relationships or two different binary genders)
   - Gay
   - Lesbian
   - Bisexual
   - Queer or pansexual
   - Two-Spirit
   - Another_____________

**Social needs**

1. **What is the highest level of education you have completed?**
   - Some grade school (grade 1-8)
   - Some high school
   - High school
   - Trades Certificate/Diploma
   - Some college/university
   - College/university degree
   - Postgraduate degree
   - No formal schooling
2. **Do you have difficulty making ends meet at the end of the month?**
   - Yes
   - No
3. **In the last 12 months, did you not fill a prescription or do anything to make a prescription last longer *because of the cost*?**
   - Yes
   - No
   - Not applicable
4. **A) What is your current housing?**
   - Own home
   - Rent
   - Staying with friends or relatives because you have no alternative [couch surfing]
   - Shelter
   - On the street
   - Other (Specify)_______________
     1. **If you rent** 🡪 **Is your current housing social housing, subsidized housing, or rent-geared-to-income?**
   - Yes
   - No
   - Not Applicable

- - 1. **If own home/rent** 🡪 **During the last 12 months, was there a time when you were not able to pay the mortgage or rent on time?**
  - Yes
  - No
  - Not applicable

1. **A) Do you feel you have family or close friends who you can open up to?**
   - Yes
   - No

**B) Are you able to rely on them if you need help (e.g. transportation, emotional or financial assistance)?**

- - Yes
  - No
  - Not applicable

1. **In the past 12 months, did you not attend an important appointment *because of the cost of transportation*?**
   - Yes
   - No
2. **In the past 12 months, did you miss making a payment on your electric, gas or other utilities bills *because of cost*?**

- Yes
- No

1. **A)** **Are you employed in a casual, short-term or temporary position?**
   - Yes
   - No

**B)** **Do you feel fearful that you could be fired if you raise employment concerns?**

- - Yes
  - No

**C)** **Does your pay vary a lot from month to month?**

- - Yes
  - No
