# Supplementary material for: “Addressing the bigger picture”: A qualitative study of internal medicine patients’ perspectives on social needs data collection and use
Source: PLoS One. 2023 Jun 7;18(6):e0285795. doi: 10.1371/journal.pone.0285795 (PMC10246844; doi:10.1371/journal.pone.0285795)
Supplement: S2 File — (DOCX) [file pone.0285795.s003.docx]

**S3 File. Interview Guide**

1. Can you tell me a bit about yourself?
2. As a reminder, I am a researcher and I do not know about your medical history because I am not part of your clinical care team. Can you tell me a bit about what led you to be admitted to the hospital?
3. Can you tell me a bit about your lifestyle and living circumstances?
4. Can you think of any challenges that you may experience following discharge from the hospital?
5. You have just filled out a set of questions asking you about your characteristics, including your race, sexual orientation, immigrant status, and language, among others. How do you feel about being asked about these characteristics while you’re in hospital?
6. Similarly, you also answered questions about your lifestyle and living circumstances, such as your social support, making ends meet, housing, and food security. How do you feel about being asked these questions in hospital?
7. Many people agree that the government helps with people’s lifestyle and living situations, such as housing and food, and that hospitals help patients with medical issues. What would it look like if hospitals helped patients with their lifestyle and living circumstances?
8. One way to help patients with financial or social need(s) is to provide them with a referral to a community organization such as a food bank, shelter or social support agency. What are your thoughts on having a hospital staff connect you to organizations to help with your need(s)?

We are considering having someone at the hospital follow-up with patients after they get a referral to an agency, to see if you accessed the services, or if you needed help accessing them. What are your thoughts on having hospital staff follow-up with you about this?

1. What are your thoughts on how your information should be used?
2. I want to talk more about your preferences on how these questions should be administered in the hospital.

- Which hospital staff members would you feel most comfortable providing this information to?
- When do you think it is best to provide this information?
- What would be the easiest way for you to provide this information?

1. Is there anything else you would like to share with me?
